# Environmental Assessment of Dryland and Irrigated Winter Wheat Cultivation under Compost Fertilization Strategies

**DOI:** 10.3390/plants13040509

**Published:** 2024-02-12

**Authors:** Elnaz Amirahmadi, Mohammad Ghorbani, Jan Moudrý, Jaroslav Bernas, Chisenga Emmanuel Mukosha, Trong Nghia Hoang

**Affiliations:** Department of Agroecosystems, Faculty of Agriculture and Technology, University of South Bohemia, Branišovská 1645/31A, 370 05 Ceske Budejovice, Czech Republic; ghorbm00@fzt.jcu.cz (M.G.); jmoudry@fzt.jcu.cz (J.M.); bernas@fzt.jcu.cz (J.B.); hoangn00@fzt.jcu.cz (T.N.H.)

**Keywords:** climate change, ecosystem quality, human health damage, LCA, resources, sustainability

## Abstract

Wheat (*Triticum aestivum* L.) is a strategic agricultural crop that plays a significant role in maintaining national food security and sustainable economic development. Increasing technical performance considering lowering costs, energy, and environmental consequences are significant aims for wheat cultivation. For drylands, which cover approximately 41% of the world’s land surface, water stress has a considerable negative impact on crop output. The current study aimed to assess the environmental aspects of chemical fertilizer in combination with compost in dryland and irrigated winter wheat production systems through life cycle assessment (LCA). The cradle-to-farm gate was considered as the system boundary based on one tone of wheat yield and four strategies: D-C (dryland with compost), D (dryland without compost), I-C (irrigated with compost), and I (irrigated without compost). Based on the results, the highest and lowest amounts of wheat yield were related to the I-C and D strategies with 12.2 and 6.7 ton ha^−1^, respectively. The LCA result showed that the I strategy in comparison with other strategies had the highest negative impact on human health (49%), resources (59%), ecosystem quality (44%), and climate change (43%). However, the D-C strategy resulted in the lowest adverse effect of 6% on human health, 1% on resources, 10% on ecosystem quality, and 11% on climate change. Utilizing a combination of fertilizer and compost in dryland areas could ensure a higher yield of crops in addition to alleviating negative environmental indicators.

## 1. Introduction

Cereals are frequently cultivated in industrialized monocultures, which are notorious for requiring enormous quantities of water, fuel, fertilizer, and pesticides, with the main objective of helping to increase production yields and, thus, best meet the world’s ever-increasing need for food [1,2,3,4]. For a large portion of the world’s population, wheat is the primary food crop, and it is the second most widely grown grain in the world [3]. The production and use of cereals are crucial since they not only greatly improve human nutrition but also pose a substantial danger to ecosystem functioning and biodiversity. Despite making up over 35% of the calories consumed by humans, cereal production typically entails damaging methods of agriculture that have the potential to drastically change the climate and deplete energy, water, and land resources [5,6].

Particularly in arid and semi-arid regions, water scarcity is a global concern that is becoming increasingly serious since it affects food insecurity [7,8,9]. Iran is one of the semi-arid countries that suffers from water scarcity. One of the best methods for increasing water production and rationalizing irrigation water has previously been described as dryland (rainfed) farming [4]. Three-quarters of the world’s population live in drylands, which make up 41% of the planet’s geographical area [10]. Dryland farming relies mostly on rainfed agriculture, in which precipitation serves as the only available supply of water for crop development [1]. However, several studies have demonstrated the detrimental effects of water stress circumstances on lowering plant metabolic activities and leaf turgor pressure in dryland farming, so crop production is significantly reduced as a result [11,12,13]. Additionally, global warming will also cause drought stress to occur more frequently in drylands, according to several studies [14,15]. As a result, improving dryland crop yields and yield stability is a major problem because of climate change and rising global food demand. On the other hand, diesel fuel is a common source of energy that is directly used by diesel engines in many agricultural activities, especially for water pumping. The use of several management techniques, such as dryland farming and nutrient management, could undoubtedly cut down on the quantity of fossil fuels required in agriculture. In other words, as an important part of agricultural activities, irrigation influences soil properties, crop growth, and the environment. Dryland farming has influenced agricultural systems by allowing growers to manage greater areas of land with reduced water and machinery inputs [1,3,16,17].

Previous research showed that adding organic amendments or fertilizers, such as composts, has been implemented for decades to maintain and enhance agricultural soil functioning [18,19,20,21,22,23]. In addition to providing crop nutrients, organic fertilizer could quickly enhance a wide range of soil characteristics, including water dynamics, nutrient cycling, and soil structure [22,24]. Among the organic fertilizers, composting has been widely accepted to produce high-quality organic fertilizer.

Life cycle assessment (LCA) is a method that is frequently used to evaluate the environmental effects of food-producing activities in the agriculture sector. With a closer look at the literature, the focus of published studies in terms of life cycle assessment in wheat cultivation is primarily on irrigated wheat farming [25,26] or dryland farming [27]. Furthermore, the evaluated factors through LCA are only based on a few environmental impact categories. To fill these knowledge gaps, a comprehensive environmental LCA of a winter wheat (*Triticum aestivum* L.) production system considering compost strategies in dryland and irrigated farming was investigated. The goal of the current study was to provide a complete evaluation of the environmental performance of compost fertilizer in both dryland and irrigated winter wheat farming systems. To fully investigate various manufacturing processes and food items, we examined several effective environmental categories in the LCA process including human health, resources, ecosystem quality, and climate change impact categories. We assumed that compost application in dryland farming could increase the dry matter accumulation and yield, as well as decrease GHG emissions and other environmental damages. The objectives of the study were to (1) determine the effects of compost fertilizer in dryland and irrigated farming on grain yield and (2) estimate the environmental damage of each cropping system. The present study’s influence is mostly related to the following aspects: the relevant number of considered impact categories, the effect of compost application on crop yield, the environmental impact of dryland and irrigated wheat farming, and, finally, the limited available literature on the topic.

## 2. Results and Discussion

### 2.1. Yield

**Figure 1:**
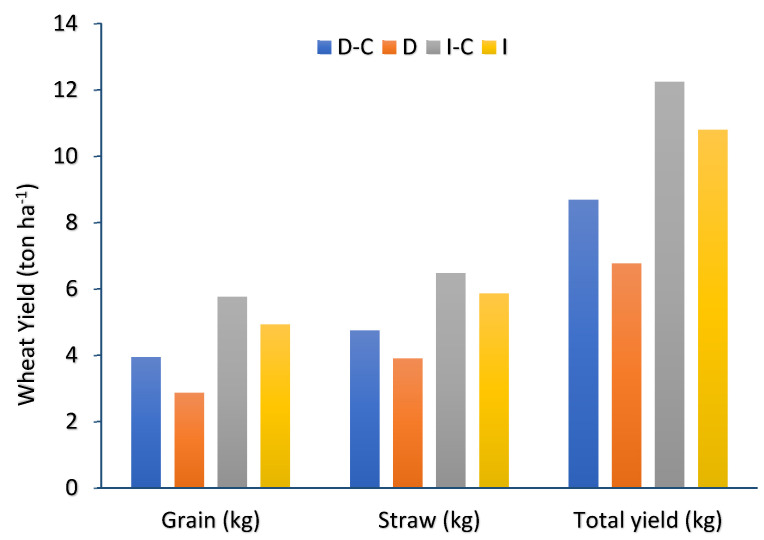
The grain, straw, and wheat yield (ton ha^−1^) in dryland and irrigated farming (D-C: dryland farming with compost, D: dryland farming without compost, I-C: irrigated farming with compost, I: irrigated farming without compost).

Applying too much or improper fertilizer does not ensure consistently high yields, which might lead to inefficient use of nutrients and numerous other issues affecting the environment and economy [28]. Therefore, learning how to improve the soil water status and optimize the coupling of fertilization and water is key to obtaining high and sustainable crop yields in arid areas. Figure 1 shows the results of wheat yield (ton ha^−1^) in different strategies. Based on the results, wheat yield (grain and straw) in irrigated farming was higher than in dryland farming, and compost fertilizer significantly affected wheat grain, straw, and total yield in both dryland and irrigated farming. In detail, the total yield of the D-C, D, I-C, and I strategies was 8.6, 6.7, 12.2, and 10.7 (ton ha^−1^), respectively. Compost fertilizer increased the wheat grain in dryland and irrigated farming by 27.4% and 14.4%, respectively. Also, wheat straw yield notably increased with the use of compost fertilizer in dryland and irrigated farming by 17.7% and 9.5%, respectively. The effect of compost application on dryland farming was higher than irrigated farming. It has been observed that under standard moisture conditions, compost application to dry soil may increase nitrogen (N) and carbon (C) mineralization [29]. Further, it was reported that compost application, both alone and combined with mineral fertilizer, improved wheat grain yield compared to the equivalent level of mineral fertilizer due to macronutrients and micronutrients in the composts. Therefore, it is obvious that when compost was given at the highest nitrogen level, the accessible phosphorus in the compost was greater than in the mineral fertilizer treatment. Furthermore, iron was only found in the compost and not in the mineral fertilizer [30]. On the other hand, the already existing organic matter in the compost substance provides an excellent habitat for microbial activity and successfully promotes nutrient transformation [31]. Consequently, a significant increase in yield in response to compost application in both dryland and irrigated is inevitable. It has been demonstrated in many countries around the world that applying organic fertilizers to agricultural soil is an effective way to regulate fertility and is the key to achieving sustained agricultural productivity.

### 2.2. Environmental Impact Assessment

**Figure 2:**
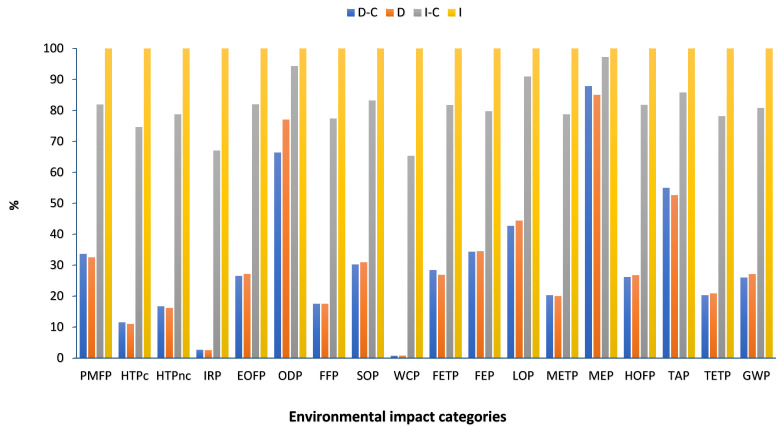
Midpoint environmental impact assessment of total yield (one ton) in dryland and irrigated wheat farming with and without compost (D-C: dryland farming with compost, D: dryland farming without compost, I-C: irrigated farming with compost, I: irrigated farming without compost).

Table 1 and Figure 2 show the impact assessment of compost fertilizer in dryland and irrigated winter wheat farming per ton. The midpoint environmental impact assessment of wheat cultivation in each strategy per ha is given in Appendix A. The values of environmental impact levels in Figure 2 were expressed in %, where 100% is the highest value within the impact categories. Based on the results, irrigated wheat farming without compost in comparison with other strategies had the highest impact categories. In detail, in dryland farming, there was a notable difference between compost and no compost strategies in PMFP, ODP, FETP, FEP, LOP, TAP, TETP, and GWP, but in irrigated farming, the significant difference was observed in all eighteen categories in terms of compost application. The results showed that compost application in both dryland and irrigated farming reduced the environmental impacts. Jiang et al. (2021) conducted a life cycle assessment of wheat production and stated that the environmental impacts of wheat production greatly improved in the manure compost strategy [32]. In addition, some research showed that compost application has many environmental benefits for improving soil quality and reducing negative environmental impact, including increasing the level of carbon storage capacity in the soil; alleviating global warming; reducing the need for fertilizers, pesticides, and peat use; improving soil structure, density, and porosity; increasing water retention capacity; and reducing erosion and nutrient leaching [33,34,35].

#### 2.2.1. Human Health

**Figure 3:**
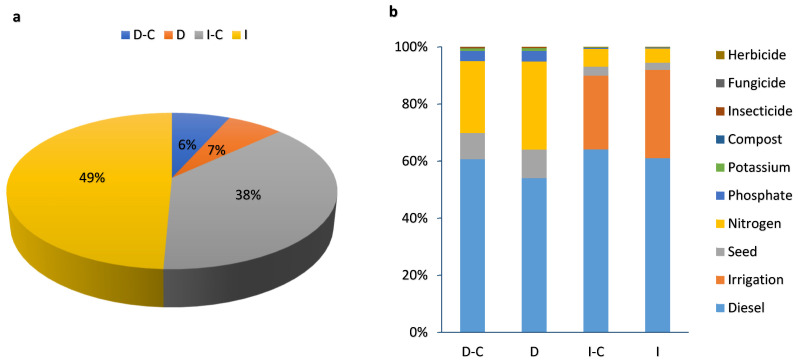
The percentage contribution of different farming strategies (**a**) and inputs (**b**) for LCA analysis in terms of human health impact category (D-C: dryland farming with compost, D: dryland farming without compost, I-C: irrigated farming with compost, I: irrigated farming without compost).

Table 1 shows the environmental impact of human health categories in dryland and irrigated winter wheat farming. The percentage contribution of dryland and irrigated wheat farming with and without compost on human health is given in Figure 3a. Irrigated farming had the highest impact on human health by 49%, but dryland compost farming, by 6%, had the lowest impact. The effect of each input on the human health of dryland and irrigated wheat farming with and without compost can be seen in Figure 3b. In dryland farming (with and without compost), diesel fuel and nitrogen fertilizer are responsible for the highest proportion of impacts on human health. On the other hand, in irrigated farming (with and without compost), diesel fuel and irrigation had the greatest impact on the human health category. It has been reported that agrochemical contamination has a long-term impact on humans, food chains, and the environment [36]. One of the issues with the use of agrochemicals is the migration of them into surface water after treatment, which can harm aquatic ecosystems and human health [37,38]. Furthermore, excessive urea fertilizer application and inadequate nitrogen usage efficiency may exacerbate these negative environmental consequences [39].

**Figure 4:**
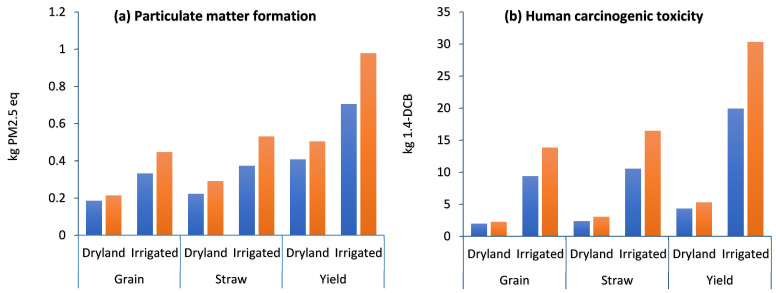

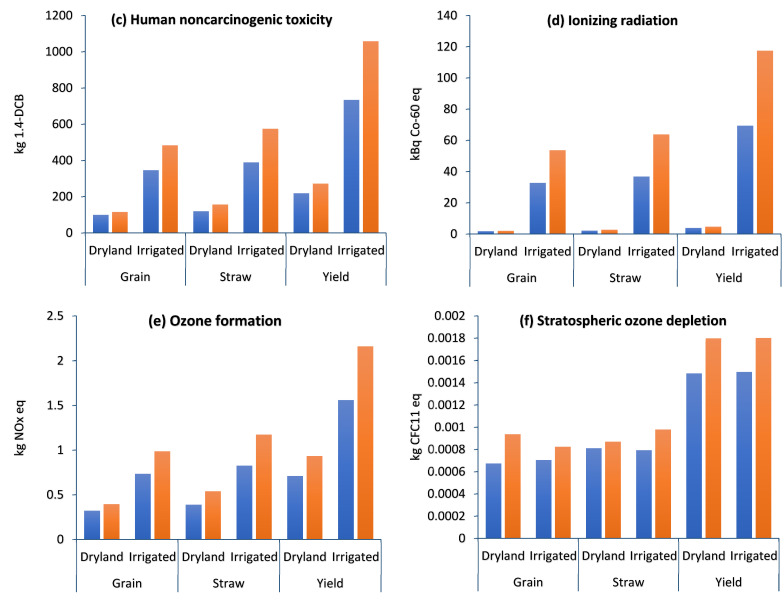
Midpoint human health impact assessment of grain, straw, and total yield (ton ha^−1^) in dryland and irrigated wheat farming with compost (blue color) and without compost (orange color).

The human health impact assessment of grain, straw, and total yield (ton ha^−1^) in dryland and irrigated wheat farming are given in Figure 4 as follows: particulate matter formation (PMFP) (Figure 4a), human carcinogenic toxicity (HTPc) (Figure 4b), human noncarcinogenic toxicity (HTPnc) (Figure 4c), ionizing radiation (IRP) (Figure 4d), ozone formation in ecosystems (EOFP) (Figure 4e), and stratospheric ozone depletion (ODP) (Figure 4f).

##### Particulate Matter Formation (PMFP)

Particulate matter (PM) is one of the major environmental pollutants and has negative effects on human health [40]. Several studies have shown that these practices affect the ecosystem and have impacts on human health [41,42,43]. The results of this research show that the PMFP in irrigated farming was higher than in dryland, and in both strategies, compost application reduced the PMEP by 23.77% and 38.72% in dryland and irrigated farming, respectively (Figure 4a). Based on the displayed results in Table 2, the share of chemical fertilizers in the compost strategy in both dryland and irrigated farming was lower than without compost strategies. Therefore, it can be a reason for the significant reduction in PMEP. On the other hand, irrigated farming relies on irrigation pumps normally powered by fossil fuels or electricity, while dryland farming relies on precipitation, resulting in lower energy consumption in this system than in irrigated farming. Also, a closer look at Table 2 shows a lower share of fossil fuel in dryland than in irrigated farming, resulting in lower emissions of air pollutants including particulates finer than 2.5 μm.

##### Human Carcinogenic Toxicity (HTPc) and Human Noncarcinogenic Toxicity (HTPnc)

The human toxicity potential (HTP) describes the potential for health damage from exposure to toxic chemicals, including carcinogens (HTPc) and noncarcinogens (HTPnc) [44,45]. In the agricultural sector, most emitted pollutants to the atmosphere are related to chemical fertilizers, pesticides, herbicides, and other agrochemicals, which play an essential role in human carcinogenic and noncarcinogenic toxicity [46,47]. The recent literature suggests that decreasing chemicals (fertilizers, herbicides, insecticides, and seed products) could be an effective technique for minimizing the human toxicity impact of wheat agriculture [48,49]. The results show that the highest HTPc and HTPnc were related to the irrigated without compost scheme and the lowest ones were related to the dryland compost farming. In detail, HTPc in the D-C, D, I-C, and I strategies was 4.31, 5.29, 19.9, and 30.3 kg 1.4-DCB, respectively (Figure 4b), while HTPnc in the D-C, D, I-C, and I strategies was 218.9, 271.3, 734.3, and 1057 kg 1.4-DCB, respectively (Table 1). In both dryland and irrigated farming, compost application reduced the HTPc and HTPnc. Darzi-Naftchali et al. reported that urea fertilizer had the largest influence on the carcinogenic diseases index [50], matching the result of current research. Table 2 shows the lower share of chemical fertilizers and biocides in compost application strategies, as a hotspot of HTP and soil emissions in both dryland and irrigated farming.

##### Ionizing Radiation (IRP)

Ionizing radiation is typically associated with traditional energy-generating operations including natural gas and petroleum extraction, coal combustion, and mining. According to the results, in both strategies, compost application reduced the IRP by 21.87% and 69.26% in dryland and irrigated farming, respectively. In detail, IRP in the D-C, D, I-C, and I strategies was 3.85, 4.69, 69.3, and 117.4 kBq Co-60 eq, respectively (Figure 4d). In cropping systems, excessive chemical fertilizer application and inadequate usage efficiency may exacerbate these negative environmental consequences [49]. On the other hand, Table 2 shows the lower share of chemical fertilizers, biocides, and diesel fuel in compost application strategies. It has been demonstrated that the use of diesel-based fossil fuels had the greatest influence on the ionizing radiation potential, followed by triple superphosphate and urea fertilizers [50]. Also, Biermann and Geist reported that agricultural machinery is the main cause of increasing ionizing radiation [51].

##### Ozone Formation (EOFP) and Stratospheric Ozone Depletion (ODP)

Heat and sunlight cause chemical reactions between oxides of nitrogen (NO_x_), and volatile organic compounds (VOC) are the main cause of ozone formation, which are also known as hydrocarbons. This reaction can occur both near the ground and high in the atmosphere. The results show that the highest and lowest EOFP were related to irrigated without compost and dryland compost farming, respectively. EOFP in the D-C, D, I-C, and I strategies was 2.15, 1.55, 0.932, and 0.709 kg NO_x_ eq, respectively (Figure 4e). In other words, compost application reduced the EOFP by 38.42% in dryland and by 31.45% in irrigated farming. The recent literature reported that approximately 90% of anthropogenic N_2_O reaches the stratosphere, thus contributing to the catalytic destruction of ozone [52,53]. As a result, the function of compost fertilizer in irrigated farming was higher than in dryland farming. The inventory data of wheat cultivation per ton (Table 2) show the higher fossil fuel consumption in irrigated farming due to the irrigation pump powered by fossil fuels in these strategies. Zheng et al. stated that agricultural machinery that operates on diesel fuel can exacerbate ozone layer depletion. Ozone-depleting compounds are a class of synthetic chemicals that cause the ozone layer’s thickness to decrease [54]. The stratospheric ozone depletion ODP in both dryland and irrigated farming was 0.002 kg CFC11 eq, and in both strategies, compost application reduced the ODP by 100%; accordingly, ODP in both dryland and irrigated farming was 0.001 kg CFC11 eq (Figure 4f). The consumption of butachlor herbicides is a source of ozone depletion. Because of the chlorine in its composition, this herbicide has high destructive power and is categorized as category one in terms of potential danger to water resources and ozone depletion [54]. Also, agricultural machinery that operates on diesel fuel can exacerbate ozone layer depletion. This claim matches the inventory data of wheat cultivation and the share of inputs in different strategies (Table 2).

#### 2.2.2. Resources

**Figure 5:**
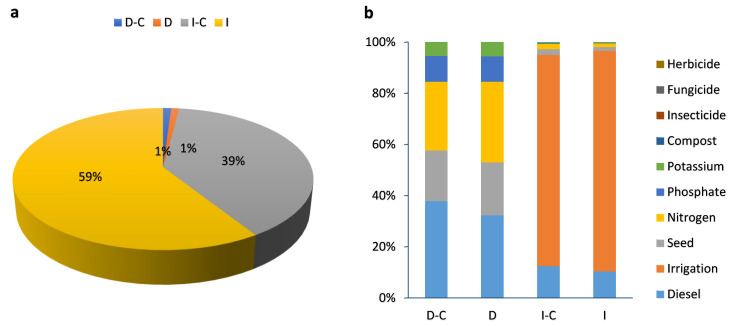
The percentage contribution of different farming strategies (**a**) and inputs (**b**) for LCA analysis in terms of resources impact category (D-C: dryland farming with compost, D: dryland farming without compost, I-C: irrigated farming with compost, I: irrigated farming without compost).

Table 1 shows the environmental impact of resource categories in dryland and irrigated winter wheat farming. The percentage contribution of dryland and irrigated wheat farming with and without compost on resources is given in Figure 5a. Irrigated farming (without compost), with a 59% contribution, had the highest impact on resources, while dryland farming both with compost and without compost fertilizer, with a 1% contribution share, had the lowest impact on resources. The effect of each input on the resource categories for dryland and irrigated wheat farming can be seen in Figure 5b. In dryland farming (with and without compost), diesel and nitrogen fertilizers are responsible for the highest proportion of impacts on resources. On the other hand, in irrigated farming, irrigation had a major impact on the resource category.

**Figure 6:**
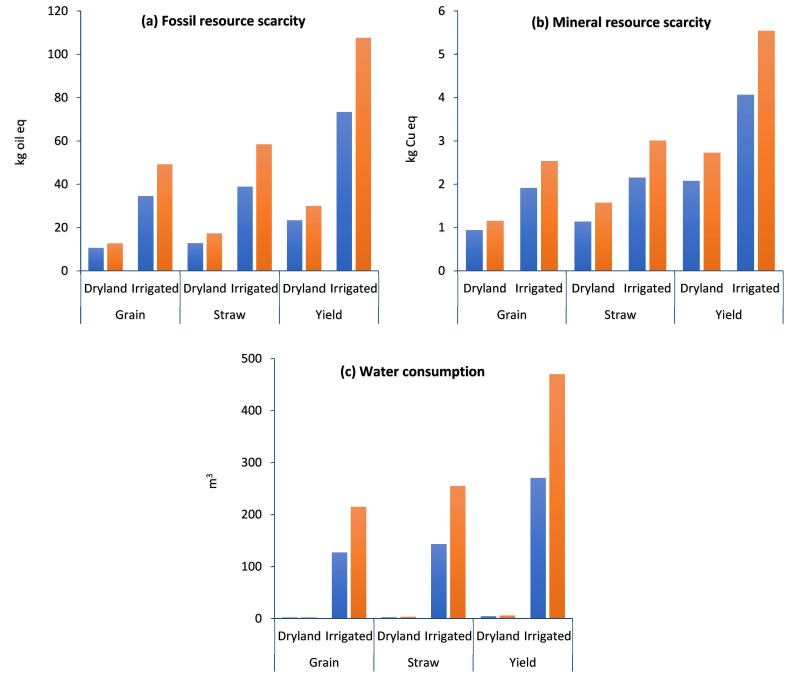
Midpoint resources impact assessment of grain, straw, and total yield (ton ha^−1^) in dryland and irrigated wheat farming with compost (blue color) and without compost (orange color).

The resource impact assessment of grain, straw, and total yield (ton ha^−1^) in dryland and irrigated wheat farming is given in Figure 6. The impact assessment of the resource categories includes fossil resource scarcity (FFP) (Figure 6a), mineral resource scarcity (SOP) (Figure 6b), and water consumption (WCP) (Figure 6c).

##### Fossil Resource Scarcity (FFP) and Mineral Resource Scarcity (SOP)

The scarcity of natural resources is a developing worry in many places in the world; on the other hand, rapid population growth and growing industrialization are putting significant strain on the world’s scarce resources, resulting in shortages in many areas [55]. Further, around 1.3–1.8% of the world’s fossil fuel consumption is attributable to the manufacturing of nitrogen fertilizers [56]. According to previous studies, chemicals such as fertilizers and insecticides account for 25–97% of all nonrenewable inputs [57]. Also, around 14% of the world’s ammonia production is based on coal gasification and 77% on natural gas reform [56]. The results show that the highest and lowest FFP was related to the irrigated without compost and dryland compost farming, respectively. Table 1 showed that FFP in the D-C, D, I-C, and I strategies was 23.37, 29.96, 73.33, and 107.5 kg oil eq, respectively. With a closer look at Table 2, it is obvious that diesel fuel and water consumption causes higher inputs in irrigated farming. Therefore, higher FFP in irrigated farming than in dryland farming is inevitable. Some studies showed that natural resource scarcity can be alleviated through conservation efforts, such as reducing water usage, improving soil fertility with appropriate amendments, and reducing energy consumption [58,59]. The result of current research shows that compost application reduced the FFP by 28.2% in dryland and by 46.4% in irrigated farming per one ton of wheat yield.

According to the results, SOP in irrigated farming was higher than in dryland, and in both strategies, compost application reduced the SOP by 31.1% and 36.3% in dryland and irrigated farming, respectively. In detail, SOP in the D-C, D, I-C, and I strategies was 2.07, 2.72, 4.06, and 5.54 kg Cu eq, respectively (Figure 5b). The use of lower amounts of fertilizers and diesel fuel in compost strategies could help to lessen the negative environmental impact on FFP and SOP.

##### Water Consumption (WCP)

The results show that the highest WCP was related to irrigated (without compost) farming with 469.8 m^3^ and the lowest WCP was related to dryland (with compost) farming with 4.407 m^3^ per ton of wheat (Table 1). Compost application reduced the WCP by 34.4% in dryland and by 73.7% in irrigated farming (Figure 5c). The impact of water depletion on individuals and ecosystems was explored under the water consumption category. WCP was calculated using m^3^ water consumption equivalents. Environmental degradation and natural resource scarcity are today’s most significant challenges. Unsustainable use of natural resources such as land, water, and air, as well as habitat degradation, are reducing the quality and availability of these resources. By improving yield and water productivity, connected irrigation and organic fertilizer management may assist farmers in coping with water shortages in drought and water-limited conditions [60]. Compost application is a reliable approach for restoring the physical and nutritional features of most soils and improving their function, particularly soils with low organic matter levels and poor structure [61]. Based on the results, the function of compost fertilizer in irrigated farming was higher than in dryland farming. Soil fertilizers, such as composting, have been demonstrated to boost soil carbon storage while also enhancing plant production and soil water-holding capacity [62]. The improvements in soil water holding capacity can have a reduction impact on drought conditions, allowing for continuous plant development even with a reduced water supply, especially in drylands [63]. It has been reported that a suitable fertilizer application can enhance water availability for crops by increasing soil water storage capacity, reducing soil evaporation, and allowing a high and sustainable crop yield [64].

#### 2.2.3. Ecosystem Quality

**Figure 7:**
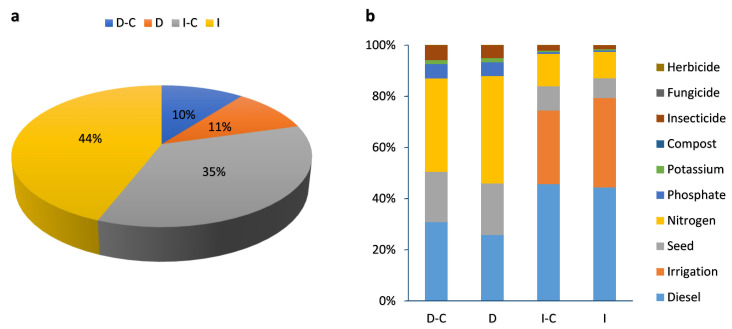
The percentage contribution of different farming strategies (**a**) and inputs (**b**) for LCA analysis in terms of ecosystem quality impact category. (D-C: dryland farming with compost, D: dryland farming without compost, I-C: irrigated farming with compost, I: irrigated farming without compost).

**Figure 8:**
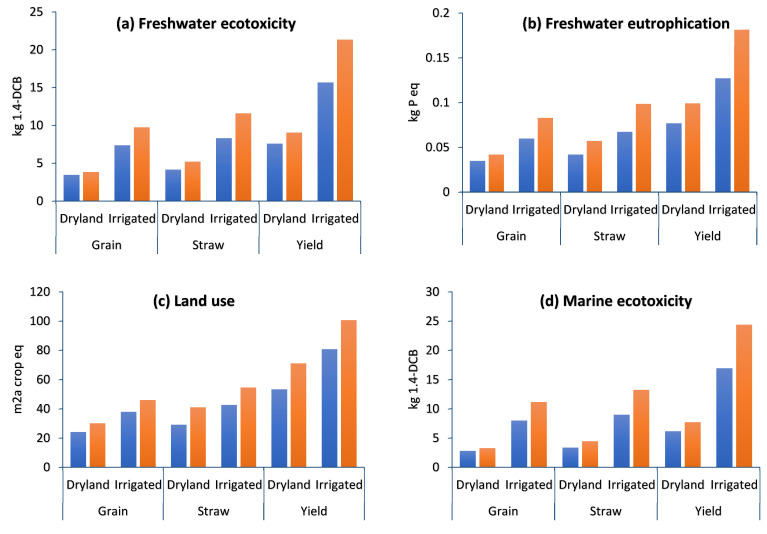

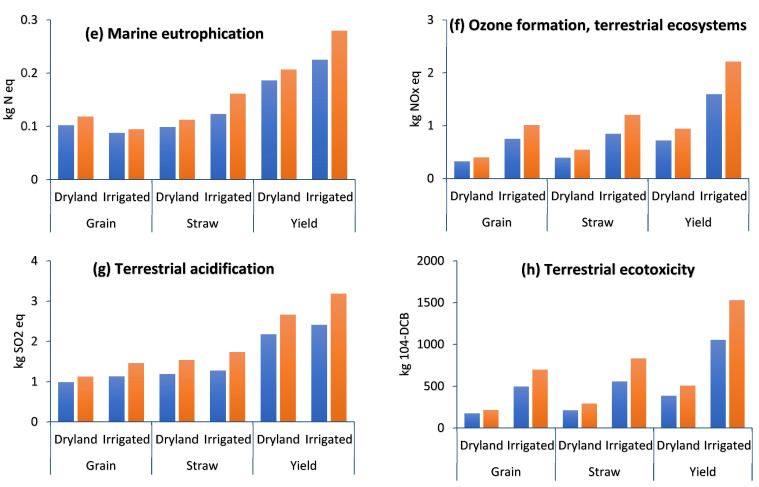
Midpoint ecosystem quality impact assessment of grain, straw, and total yield (ton ha^−1^) in dryland and irrigated wheat farming with compost (blue color) and without compost (orange color).

The percentage contribution of different wheat farming strategies on ecosystem quality is given in Figure 7a. Table 1 and Figure 8 show the ecosystem quality categories in dryland and irrigated winter wheat farming. Irrigated farming (without compost), by 44%, had the highest impact on ecosystem quality, but dryland farming (with compost), by 10%, had the lowest impact on ecosystem quality. The effect of each input on the ecosystem quality for dryland and irrigated wheat farming can be seen in Figure 7b. In dryland farming (with and without compost), nitrogen fertilizer and diesel fuel are responsible for the highest proportion of impacts on ecosystem quality. On the other hand, in irrigated farming (with and without compost), diesel fuel and irrigation had the greatest impact on the ecosystem quality category.

The ecosystem quality impact assessment of grain, straw, and total yield (ton ha^−1^) in dryland and irrigated wheat farming is given in Figure 8. The impact assessment of ecosystem quality categories includes freshwater ecotoxicity (FETP) (Figure 8a), freshwater eutrophication (FEP) (Figure 8b), land use (LOP) (Figure 8c), marine ecotoxicity (METP) (Figure 8d), marine eutrophication (MEP) (Figure 8e), ozone formation in terrestrial ecosystems (HOFP) (Figure 8f), terrestrial acidification (TAP) (Figure 8g), and terrestrial ecotoxicity (TETP) (Figure 8h).

##### Freshwater Ecotoxicity (FETP) and Freshwater Eutrophication (FEP)

Groundwater contamination and freshwater ecotoxicity are caused by the leaching of soil contaminants and toxic substances that affect the quality of surface water [65]. However, eutrophication is caused by excessive phytoplankton development on surface waters because of the availability of nutrients such as phosphorous and nitrogen derivative compounds, which causes the loss of both ecosystem health and aquatic biodiversity. Chemical pollution’s impact on freshwater ecosystems not only leads to a direct impact on aquatic life but also decreases their potential for producing ecosystem services in ways that negatively affect human well-being, creating a challenge to sustainable ecosystem service production [66]. The results show that FETP in the D-C, D, I-C, and I strategies was 7.58, 9.03, 15.65, and 21.32 kg 1.4-DCB, respectively (Figure 8a), while FEP in the D-C, D, I-C, and I strategies was 0.07, 0.09, 0.12, and 0.18 kg P eq, respectively (Figure 8b). FETP in dryland farming was lower than in irrigated farming. The results showed that compost application reduced FETP by 19.1% in dryland and 36.2% in irrigated farming. The toxicity impact is mostly caused by the emission of nitrogen dioxide (NO_2_) into the air or soil [67], which is significantly reduced by compost application in both dryland and irrigated farming (Table 2 and Table 3). Soil toxicity is related to the use of fertilizers and chemical pesticides in agriculture, and soil is a significant pollution sink due to particle adsorption on the soil surface [68]. Compost is used as a natural soil nourishment or organic fertilizer as a substitute for synthetic fertilizers, whose unregulated usage has resulted in negative environmental problems, loss of various soil qualities, and crop yields over time [53]. Also, previous research reported that during crop cultivation, the use of fertilizer leads to higher eutrophication potential [3,69,70].

##### Land Use (LOP)

The impact category ‘land use’ describes the environmental impacts of occupying, reshaping, and managing land for human purposes. Land use can either be the long-term use of land (e.g., for arable farming) or changing the type of land use (e.g., from natural to urban area). LOP is expressed in (m^2^ × yearly) crop equivalents [53]. According to the results, LOP in irrigated farming was higher than in dryland, and in both strategies, compost application reduced the LOP by 33.4% and 24.6% in dryland and irrigated farming, respectively. In detail, LOP in the D-C, D, I-C, and I strategies was 53.29, 71.12, 80.65, and 100.5 m^2^a crop eq, respectively (Figure 8c). In this study, the total yield of the D-C, D, I-C, and I strategies was 8.6, 6.7, 12.2, and 10.7 (ton ha^−1^), respectively, and compost fertilizer increased the wheat grain of dryland and irrigated farming by 27.4% and 14.4%, respectively. Therefore, to produce the same amount of wheat, less land is required. This emphasized that in order to achieve equivalent yields with chemical fertilizer, compost application requires a lower surface area of land and also reduces the agricultural machinery.

##### Marine Ecotoxicity (METP) and Marine Eutrophication (MEP)

In general, human activities continue to put strain on marine ecosystems, such as pollution from chemicals generated through manufacturing cycles [71]. Marine ecotoxicity is the effect of harmful compounds emitted into the environment on marine creatures, but eutrophication is the unwanted increase in biomass in ecosystems caused by nutrient enrichment, and fertilizer emissions of nitrogen and phosphorus are a key contribution to eutrophication [56]. The results show that METP in the D-C, D, I-C, and I strategies was 6.16, 7.71, 16.9, and 24.3 kg 1.4-DCB, respectively (Figure 8d). Irrigation contributes to nitrate leaching; therefore, irrigation water promotes absorption through the soil profile, which may result in the transfer of soluble nitrogen, mostly in the form of nitrate [72]. If the amount of irrigation water used exceeds the soil’s water-holding capacity, nitrate could leach beyond the root zone, which may cause marine ecotoxicity and water pollution [73,74]. In detail, compost application reduced METP, by 25.2% and 10.7%, in both dryland and irrigated farming, respectively, owing to lower diesel fuel consumption and synthetic fertilizer use (Table 2). Leachate can pollute both groundwater and surface water as the main reason for marine ecotoxicity. According to the results, compost application reduced MEP, by 26.2% and 10.7%, in dryland and irrigated farming, respectively, owing to lower levels of agrochemicals and synthetic fertilizer use (Table 2). Prechsl et al. found that heavy metal emissions from fertilizers had the greatest influence on the ecosystem [75]; on the other hand, organic fertilizers have a lower impact on eutrophication due both to the lower quantity used and to the formation of N, which is organic. Fertilizer emissions of nitrogen and phosphorus are a key contribution to eutrophication. According to the previous reports, in terms of eutrophication potential, phosphorus fertilizers and pesticides had a much larger percentage than other input sources. Also, the use of nitrogen fertilizer has a significant impact on the eutrophication of water sources [56,68,70].

##### Ozone Formation in Terrestrial Ecosystems (HOFP), Terrestrial Acidification (TAP), and Terrestrial Ecotoxicity (TETP)

The results show that the highest ozone formation in terrestrial ecosystems (HOFP) was related to irrigated farming (without compost) by 2.21 kg NO_x_ eq and the lowest HOFP was related to dryland (with compost) by 0.71 kg NO_x_ eq per ton wheat (Figure 8f). Compost application reduced the HOFP by 31.3% in dryland and by 38.6% in irrigated farming. It is obvious that agrochemicals are being used to reduce some undesirable organisms to increase crop productivity. However, certain agrochemicals may have an influence on humans as well as aquatic and terrestrial ecosystems through leaching, evaporation, and surface run-off [76,77]. Terrestrial acidification (TAP) is being calculated using kg SO_2_ equivalents emitted into the atmosphere [77]. Soil acidity changes are caused by atmospheric deposition of inorganic chemicals, such as sulfates, nitrates, and phosphates, and have an adverse effect on all plant species and soil microorganisms, while terrestrial ecotoxicity considers the potential damage and hazardous effects caused to terrestrial ecosystems by releasing chemicals into the environment [76].

According to the results, TAP and TETP in irrigated farming were higher than in dryland. In detail, TAP in the D-C, D, I-C, and I strategies was 2.17, 2.65, 2.41, and 3.18 kg SO_2_ eq, respectively (Figure 8g), and TETP in the D-C, D, I-C, and I strategies was 384.8, 506.9, 1053, and 1529 kg 104-DCB, respectively (Figure 8h). The results showed that compost application reduced the TAP by 22.31% and 32.38% in dryland and irrigated farming, respectively. Organic fertilizers, in general, have a low potential to acidify the soil. Chemical fertilizers, on the other hand, have varying qualities depending on their composition. Also, TETP was reduced under compost application by 31.72% in dryland and 45.21% in irrigated farming. Crop cultivation influences acidification and ecotoxicity due to the production of the chemicals used, transportation of raw materials, and energy generation for the process. The result of the current research shows that compost application reduced the inputs (agrochemicals and diesel fuel consumption) in both dryland and irrigated farming (Table 2), and it aligned with the lowest TAP and TETP in compost application strategies. Studies have shown that the unbalanced and excessive use of mineral and chemical fertilizers decreases crop yield and soil physical characteristics and increases nitrate and heavy metal accumulation, as well as soil acidity [33,78]. It has been reported that fertilization treatments have a significant influence on terrestrial ecotoxicity. By evaluating various fertilization procedures, Krzyżaniak et al. discovered that decreasing fertilization reduced terrestrial ecotoxicity [79].

#### 2.2.4. Climate Change and Global Warming (GWP)

**Figure 9:**
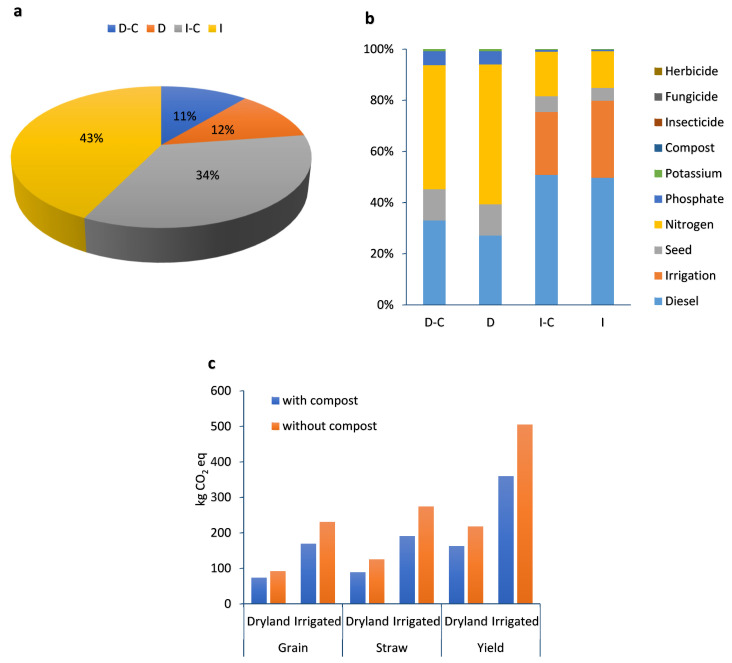
The percentage contribution of different farming strategies (**a**) inputs (**b**), and midpoint global warming impact assessment of grain, straw, and total yield (**c**) (ton ha^−1^) (D-C: dryland farming with compost, D: dryland farming without compost, I-C: irrigated farming with compost, I: irrigated farming without compost).

Table 1 shows the global warming potential of dryland and irrigated winter wheat farming. Also, the global warming potential of grain, straw, and total yield (ton ha^−1^) are given in Figure 9c. The percentage contribution of dryland and irrigated wheat farming to climate change is given in Figure 9a. Based on the results, irrigated farming (without compost), with a contribution share of 43%, had the highest impact on climate change, while dryland (with compost) farming, with an 11% contribution, had the lowest impact on climate change. According to Wang et al. deficit irrigation significantly reduced greenhouse gas emissions from winter wheat fields when compared to full irrigation due to the lower fossil fuel consumption [80]. Also, Zornoza et al. showed that irrigation increases N_2_O and CO_2_ emissions compared to no irrigation because of an enhancement in soil respiration, soil-accessible water, and carbon and nitrogen mineralization (Zornoza et al., 2018) [81].

The effect of each input on climate change for dryland and irrigated wheat farming with and without compost can be seen in Figure 9b. In dryland farming, nitrogen fertilizer and diesel fuel are responsible for the highest proportion of impacts on climate change, since fertilizer production uses a lot of coal or natural gas as a source of hydrogen (to synthesize ammonia), resulting in increasing CO_2_ emissions. On the other hand, in irrigation farming (with and without compost), diesel fuel and irrigation (relying on an irrigation pump powered by fossil fuels) had a major impact on climate change. According to Hokazono and Hayashi, in the rice system, direct emissions and field operations significantly contribute to the environmental impacts [82]. Similar research studies showed that the usage of chemical fertilizers (especially urea) and fossil fuels had the greatest impact on global warming potential and GHG emissions in other crops [28]. The global warming potential (GWP) values found for the D-C, D, I-C, and I strategies were 162.6, 217.6, 359.7, and 504.8 kg CO_2_ eq per one ton of total yield, respectively (Table 1). The results showed that compost application reduced the GWP by 33.8% and 40.3% in dryland and irrigated farming, respectively. Compost application can boost soil carbon stocks directly by adding organic material and indirectly by improving soil C sequestration through plant inputs [83]. In other words, compost fertilizer improves water-holding capacity, and the slow-release nutrients in compost promote carbon sequestration through photosynthesis after which carbon is incorporated into plant biomass [33].

## 3. Materials and Methods

### 3.1. Study Site and Data Collection

This study was conducted in Hamedan state of Iran, which is located at 33°59′–35°48′ N, and 47°34′–49°36′ E. This region had a cold semi-arid climate (Koppen climate classification BSk) with an average rainfall of 33.9 and 51 mm and an average annual temperature of 13.3 and 11.7 °C. Soil chemical and physical properties in the study area are given in Table S1. The data were collected using questionnaires and interviews from 88 farms. The appropriate sample number for this study was determined using Cochran’s formula and using a simple random sampling technique:

$$n=\frac{N{\left(s\times t\right)}^{2}}{\left(N-1\right)d^{2}+N{\left(s\times t\right)}^{2}}$$

where *n* is the required sample number, *N* is the population size, *t* is the *t* value at 95% confidence limit (1.96), *s* is the standard deviation (SD), and *d* is the permissible error (5%).

Four strategies were considered for this research: D-C (dryland with compost), D dryland without compost), I-C (irrigated with compost), and I (irrigated without compost). Table S2 indicates the fundamental properties of each strategy.

### 3.2. Life Cycle Assessment

The LCA methodology of this study is based on the ISO 14040 and ISO 14044 standards and contains four phases, including goal and scope definition, life cycle inventory (LCI), life cycle impact assessment (LCIA), and data interpretation. In the current research, the environmental impact of compost fertilizer in dryland and irrigated farming was evaluated. The LCA of this study is conducted with the ReCiPe 2016 midpoint (H) impact assessment, AGRIBALYSE v1.2/v1.3, and Ecoinvent V.3.5 databases. OpenLCA software V.11.0 was used to assess the environmental impact of compost fertilizer in dryland and irrigated farming.

**Figure 10:**
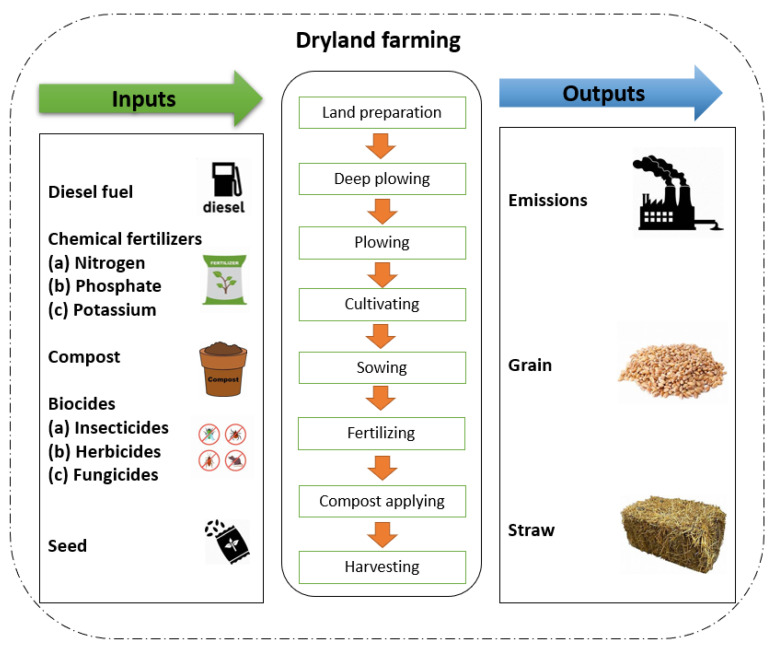

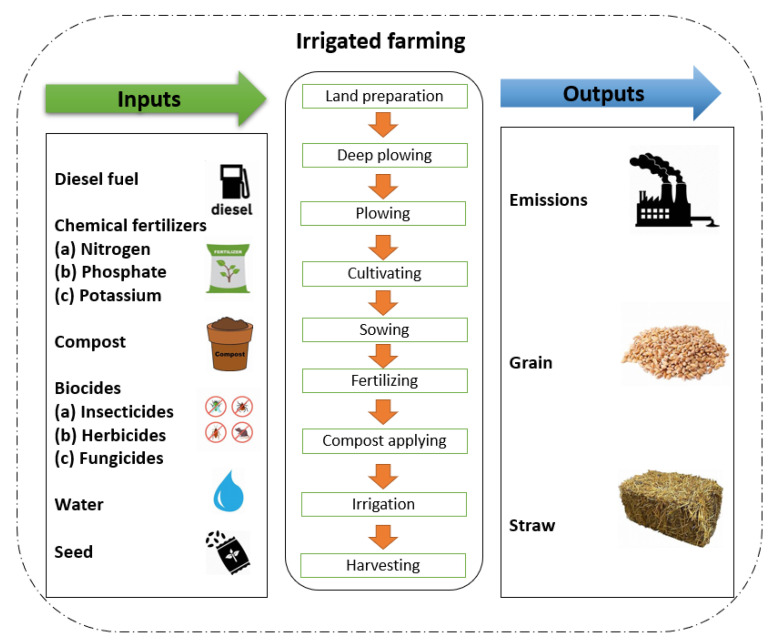
System boundaries of dryland and irrigated wheat farming.

The goal and scope are the first phase of any LCA research and consist of the functional unit, system boundary, and study aim. As a comparative condition, the functional unit is connected to the inputs and outputs. Several operations are required for wheat cultivation, i.e., land preparation, deep plowing, plowing, cultivation, sowing, fertilizing, compost application, irrigation, and harvesting. One tone of dry matter was considered as the functional unit for this research. Also, the system boundary of cradle-to-farm gate was considered in this research, (Figure 10). The emissions and nutrient losses were evaluated based on the following terms: carbon dioxide (CO_2_), nitrous oxide (N_2_O), nitrates (NO_3_), nitric oxide (NO), nitrogen dioxide (NO_2_), ammonia (NH_3_), nitrogen (N), phosphates (PO_4_), and phosphorus (P).

In the second phase, life cycle inventory (LCI) includes information on all environmental inputs and outputs at each step of the life cycle. The LCI was constructed using two datasets including foreground and background. Foreground data for this study were obtained through farmer interviews, and the sources of the background data were AGRIBALYSE v1.2/v1.3 and Ecoinvent V.3.5 databases. The inventory data of wheat cultivation in each strategy are shown in Table 2 (per ton) and Table 3 (per hectare)

**Figure 11:**
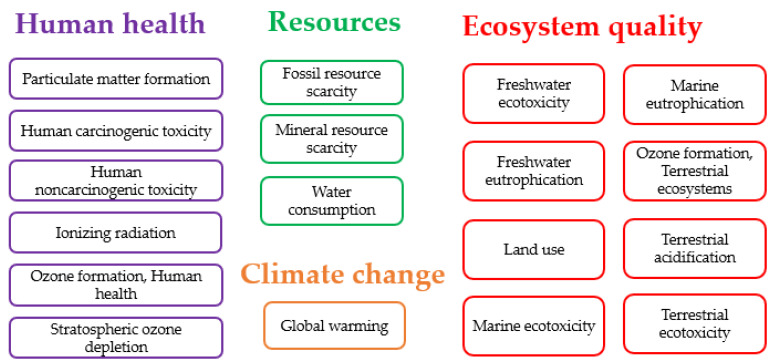
Overview of the midpoint impact categories in four different damage categories (human health, ecosystem quality, resources, and climate change).

In the next step, classification, characterization, normalization, and weighting were four categories of the life cycle impact assessment (LCIA) as the third phase of LCA. The ReCiPe 2016 model was used for the assessment of the environmental impact in this study. The overall purpose of the ReCiPe method is to condense a lengthy list of life cycle inventory data into a small number of indicator scores. The ReCiPe midpoint technique includes 18 environmental impact indicators, which are further divided into four damage categories (human health, ecosystem quality, resources, and climate change) (Figure 11).

Finally, the goal of data interpretation as the fourth phase of LCA was to analyze the findings to discover the restrictions, suggestions, and results of the LCA to assist decision makers.

## 4. Conclusions

Water shortages have shown detrimental impacts on crop yield. This problem could be overcome by applying organic amendments and fertilizers. In the current research, wheat yield and environmental indicators of compost fertilization in dryland and irrigated wheat production were compared from multi-environmental aspects using the life cycle assessment (LCA) technique. In both dryland and irrigated farming, compost application increased the amount of yield due to providing a sustainable source of essential nutrients for plant growth. In general, the impact of compost fertilization on grain yield in dryland farming was higher than in irrigated farming. Also, the application of compost notably reduced the environmental indicators in both farming systems. The lowest environmental impacts of compost application in dryland and irrigated farming showed the outstanding potential of compost as an organic soil fertilizer, in terms of securing human health, resources, ecosystem quality, and net-zero emissions. Therefore, it would be suggested that utilizing a combination of fertilizer and compost, especially in dryland areas, could ensure a higher yield while also alleviating the negative environmental indicators.

## Figures and Tables

**Table 1:** Midpoint environmental impact assessment of wheat cultivation in each strategy per ton.

Damage Category	Impact Category	Abbreviation	Unit	Dryland		Irrigated	
				With Compost	Without Compost	With Compost	Without Compost
Human health	Particulate matter formation	PMFP	kg PM2.5 eq	0.408 ^d^	0.505 ^c^	0.705 ^b^	0.978 ^a^
	Human carcinogenic toxicity	HTPc	kg 1.4-DCB	4.314 ^c^	5.297 ^c^	19.92 ^b^	30.3 ^a^
	Human noncarcinogenic toxicity	HTPnc	kg 1.4-DCB	218.9 ^c^	271.3 ^c^	734.3 ^b^	1057 ^a^
	Ionizing radiation	IRP	kBq Co-60 eq	3.854 ^c^	4.697 ^c^	69.36 ^b^	117.4 ^a^
	Ozone formation	EOFP	kg NO_x_ eq	0.709 ^c^	0.932 ^c^	1.559 ^b^	2.158 ^a^
	Stratospheric ozone depletion	ODP	kg CFC11 eq	0.001 ^b^	0.002 ^a^	0.001 ^b^	0.002 ^a^
Resources	Fossil resource scarcity	FFP	kg oil eq	23.37 ^c^	29.96 ^c^	73.33 ^b^	107.5 ^a^
	Mineral resource scarcity	SOP	kg Cu eq	2.076 ^c^	2.726 ^c^	4.064 ^b^	5.542 ^a^
	Water consumption	WCP	m^3^	4.407 ^c^	5.927 ^c^	270.4 ^b^	469.8 ^a^
Ecosystem quality	Freshwater ecotoxicity	FETP	kg 1.4-DCB	7.582 ^d^	9.034 ^c^	15.65 ^b^	21.32 ^a^
	Freshwater eutrophication	FEP	kg P eq	0.077 ^c^	0.099 ^c^	0.127 ^b^	0.181 ^a^
	Land use	LOP	m^2^ a crop eq	53.29 ^d^	71.12 ^c^	80.65 ^b^	100.5 ^a^
	Marine ecotoxicity	METP	kg 1.4-DCB	6.162 ^c^	7.719 ^c^	16.95 ^b^	24.38 ^a^
	Marine eutrophication	MEP	kg N eq	0.225 ^a^	0.284 ^a^	0.186 ^b^	0.206 ^a^
	Ozone formation, terrestrial ecosystems	HOFP	kg NO_x_ eq	0.717 ^c^	0.942 ^c^	1.595 ^b^	2.212 ^a^
	Terrestrial acidification	TAP	kg SO_2_ eq	2.174 ^c^	2.659 ^b^	2.406 ^b^	3.185 ^a^
	Terrestrial ecotoxicity	TETP	kg 1.4-DCB	384.8 ^d^	506.9 ^c^	1053 ^b^	1529 ^a^
Climate change	Global warming	GWP	kg CO_2_ eq	162.6 ^d^	217.6 ^c^	359.7 ^b^	504.8 ^a^

The lower-case characters represent significant differences between strategies, while the same letters indicate no significant difference between strategies.

**Table 2:** The inventory data of wheat cultivation in each strategy per ton.

		Dryland		Irrigated	
		With Compost	Without Compost	With Compost	Without Compost
Inputs	1. Human labor (h)	17.65	22.18	13.09	15.24
	2. Machinery (h)	1.386	1.587	5.502	7.676
	(a) Tractor, land preparation (h)	0.368	0.443	0.229	0.232
	(b) Moldboard plow, plowing (h)	0.138	0.177	0.082	0.093
	(b) Disc plow, plowing (h)	0.144	0.185	0.090	0.102
	(d) Field cultivator, cultivating (h)	0.173	0.221	0.110	0.125
	(e) Broadcaster, sowing (h)	0.104	0.133	0.065	0.074
	(f) Broadcaster, fertilizing (h)	0.058	0.074	0.041	0.046
	(g) Tractor, compost applying (h)	0.127	0.000	0.090	0.000
	(g) Irrigation (h)	0.000	0.000	4.599	6.782
	(h) Combine harvester, harvesting (h)	0.276	0.354	0.196	0.222
	3. Diesel fuel (L)	5.03	5.55	19.25	26.86
	4. Chemical fertilizers (kg)				
	(a) Nitrogen (N)	6.746	10.25	6.016	7.079
	(b) Phosphate (P_2_O_5_)	8.322	10.58	2.819	3.144
	(c) Potassium (K_2_O)	1.210	1.615	1.170	1.284
	6. Organic fertilizer (kg)				
	Compost	345.11	0.00	245.06	0.00
	7. Biocides (kg)				
	Insecticides	0.019	0.021	0.018	0.019
	Herbicides	0.020	0.027	0.094	0.106
	Fungicides	0.019	0.024	0.025	0.031
	8. Water (m^3^)	0.0	0.0	262.6	459.3
	9. Seed (kg)	15.58	21.01	19.51	22.73
Outputs	CO_2_ (kg eq. CO_2_)	51.5	68.0	83.2	110.2
	N_2_O (kg N_2_O)	0.295	0.264	0.250	0.198
	NO_3_ (kg NO_3-_ N)	2.512	3.192	1.785	1.993
	NO (kg NO)	0.288	0.409	0.236	0.283
	NO_2_ (kg NO_2_)	0.288	0.410	0.237	0.283
	NH_3_ (kg NH_3_)	0.783	0.921	0.477	0.558
	N (kg N)	0.188	0.168	0.159	0.126
	PO_4_ (kg PO_4_)	0.002	0.002	0.001	0.001
	P (kg P)	0.046	0.059	0.026	0.030
	Grain (kg)	453.5	423.3	470.5	457.0
	Straw (kg)	546.5	576.7	529.5	543.0

**Table 3:** The inventory data of wheat cultivation in each strategy per ha.

		Dryland		Irrigated	
		With Compost	Without Compost	With Compost	Without Compost
Inputs	1. Human labor (h)	153.4	150.2	160.3	164.5
	2. Machinery (h)	12.05	10.75	67.35	82.85
	(a) Tractor, land preparation (h)	3.2	3	2.8	2.5
	(b) Moldboard plow, plowing (h)	1.2	1.2	1	1
	(b) Disc plow, plowing (h)	1.25	1.25	1.1	1.1
	(d) Field cultivator, cultivating (h)	1.5	1.5	1.35	1.35
	(e) Broadcaster, sowing (h)	0.9	0.9	0.8	0.8
	(f) Broadcaster, fertilizing (h)	0.5	0.5	0.5	0.5
	(g) Tractor, compost applying (h)	1.1	0	1.1	0
	(g) Irrigation (h)	0	0	56.3	73.2
	(h) Combine harvester, harvesting (h)	2.4	2.4	2.4	2.4
	3. Diesel fuel (L)	43.75	37.62	235.7	289.9
	4. Chemical fertilizers (kg)				
	(a) Nitrogen (N)	58.64	69.42	73.65	76.41
	(b) Phosphate (P_2_O_5_)	72.34	71.65	34.51	33.94
	(c) Potassium (K_2_O)	10.52	10.94	14.32	13.86
	6. Organic fertilizer (kg)				
	Compost	3000	0	3000	0
	7. Biocides (kg)				
	Insecticides	0.161	0.144	0.215	0.201
	Herbicides	0.175	0.182	1.154	1.149
	Fungicides	0.165	0.161	0.312	0.335
	8. Water (m^3^)	0	0	3215	4958
	9. Seed (kg)	135.4	142.3	238.9	245.4
Outputs	CO_2_ (kg eq. CO_2_)	447.3	460.6	1019.1	1189.8
	N_2_O (kg N_2_O)	2.564	1.785	3.055	2.133
	NO_3_ (kg NO_3-_ N)	21.84	21.62	21.85	21.51
	NO (kg NO)	2.502	2.771	2.891	3.053
	NO_2_ (kg NO_2_)	2.504	2.777	2.897	3.056
	NH_3_ (kg NH_3_)	6.805	6.237	5.843	6.018
	N (kg N)	1.631	1.135	1.944	1.357
	PO_4_ (kg PO_4_)	0.018	0.016	0.013	0.012
	P (kg P)	0.403	0.401	0.320	0.319
	Grain (kg)	3942	2867	5760	4933
	Straw (kg)	4751	3906	6482	5861
	Yield (kg)	8693	6773	12,242	10,794

## Data Availability

Dataset available on request from the authors.

## Supplementary Materials

The following supporting information can be downloaded at: https://www.mdpi.com/article/10.3390/plants13040509/s1, Table S1: Characteristics of soil in the studied site before the experiment. Table S2: Fundamental properties of the production systems.

## Nomenclature

Four strategies in the study	
D-C	Dryland farming with compost
D	Dryland farming without compost
I-C	Irrigated farming with compost
I	Irrigated farming without compost
LCA	Life cycle assessment
LCI	Life cycle inventory
PMFP	Particulate matter formation
HTPc	Human carcinogenic toxicity
HTPnc	Human noncarcinogenic toxicity
IRP	Ionizing radiation
EOFP	Ozone formation
ODP	Stratospheric ozone depletion
FFP	Fossil resource scarcity
SOP	Mineral resource scarcity
WCP	Water consumption
FETP	Freshwater ecotoxicity
FEP	Freshwater eutrophication
LOP	Land use
METP	Marine ecotoxicity
MEP	Marine eutrophication
HOFP	Ozone formation, terrestrial ecosystems
TAP	Terrestrial acidification
TETP	Terrestrial ecotoxicity
GWP	Global warming

## Appendix A

**Table A1:** Midpoint environmental impact assessment of wheat cultivation in each strategy per ha.

Damage Category	Impact Category	Abbreviation	Unit	Dryland		Irrigated	
				With Compost	Without Compost	With Compost	Without Compost
Human health	Particulate matter formation	PMFP	kg PM2.5 eq	3.545 ^c^	3.427 ^c^	8.635 ^b^	10.55 ^a^
	Human carcinogenic toxicity	HTPc	kg 1.4-DCB	37.51 ^c^	35.88 ^c^	243.9 ^b^	327.1 ^a^
	Human noncarcinogenic toxicity	HTPnc	kg 1.4-DCB	1903 ^c^	1838 ^c^	8984 ^b^	11,413 ^a^
	Ionizing radiation	IRP	kBq Co-60 eq	33.51 ^c^	31.82 ^c^	849.2 ^b^	1267 ^a^
	Ozone formation	EOFP	kg NO_x_ eq	6.167 ^c^	6.317 ^c^	19.08 ^b^	23.29 ^a^
	Ozone formation	ODP	kg CFC11 eq	0.013 ^b^	0.015 ^b^	0.018 ^a^	0.019 ^a^
Resources	Fossil resource scarcity	FFP	kg oil eq	203.2 ^c^	202.9 ^c^	897.8 ^b^	1161 ^a^
	Mineral resource scarcity	SOP	kg Cu eq	18.05 ^c^	18.46 ^c^	49.75 ^b^	59.82 ^a^
	Water consumption	WCP	m^3^	38.29 ^c^	40.14 ^c^	3310 ^b^	5071 ^a^
Ecosystem quality	Freshwater ecotoxicity	FETP	kg 1.4-DCB	65.04 ^c^	61.55 ^c^	187.4 ^b^	229.3 ^a^
	Freshwater eutrophication	FEP	kg P eq	0.671 ^c^	0.673 ^c^	1.558 ^b^	1.954 ^a^
	Land use	LOP	m^2^a crop eq	463.1 ^c^	481.7 ^c^	987.6 ^b^	1085 ^a^
	Marine ecotoxicity	METP	kg 1.4-DCB	53.27 ^c^	52.41 ^c^	206.9 ^b^	262.9 ^a^
	Marine eutrophication	MEP	kg N eq	1.956 ^b^	1.894 ^b^	2.278 ^a^	2.228 ^a^
	Ozone formation, terrestrial ecosystems	HOFP	kg NO_x_ eq	6.238 ^c^	6.382 ^c^	19.52 ^b^	23.87 ^a^
	Terrestrial acidification	TAP	kg SO_2_ eq	18.89 ^c^	18.07 ^c^	29.46 ^b^	34.37 ^a^
	Terrestrial ecotoxicity	TETP	kg 1.4-DCB	3341 ^c^	3436 ^c^	12,890 ^b^	16,503 ^a^
Climate change	Global warming	GWP	kg CO_2_ eq	1413 ^c^	1474 ^c^	4401 ^b^	5449 ^a^

The lower-case characters represent significant differences between strategies, while the same letters indicate no significant difference between strategies.
