# Supplementary material for: Environmental Assessment of Dryland and Irrigated Winter Wheat Cultivation under Compost Fertilization Strategies
Source: Plants (Basel). 2024 Feb 12;13(4):509. doi: 10.3390/plants13040509 (PMC10893302; doi:10.3390/plants13040509)
Supplement: Supplementary file 1 [file plants-13-00509-s001.zip › plants-2854018-supplementary.pdf]

Table S1. Characteristics of soil in the studied site before the experiment.

Physicochemical parameters	Dryland	Irrigated
pH	7.92 ± 0.06	8.12 ± 0.05
EC (dS m^{-1})	0.51 ± 0.03	0.59 ± 0.04
OC (%)	0.65 ± 0.16	0.76 ± 0.03
N (%)	0.06 ± 0.003	0.09 ± 0.001
P (mg kg^{-1})	8.59 ± 0.29	9.12 ± 0.06
K (mg kg^{-1})	312 ± 6.32	354 ± 7.12
CEC ($cmol^{(+)}$ kg^{-1}soil)	39.3 ± 0.9	39.6 ± 0.9
Bulk density	1.49 ± 0.04	1.23 ± 0.09
Sand: Silt: Clay (%)	33.1: 38.5: 28.4	19.9: 49.2: 30.9
Soil texture	Clay loam	Silty clay loam

EC: electrical conductivity, OC: organic matter, N: nitrogen, P: phosphorus, K: potassium, CEC: cation exchangeable capacity.

Table S2. Fundamental properties of the production systems.

	Strategy 1	Strategy 2	Strategy 3	Strategy 4
	**Dryland**		**Irrigated**	
	**D-C**	**D**	**I-C**	**I**
System of nutrient management	NPK + Compost	NPK	NPK + Compost	NPK
Place of cultivation	Hamedan state of Iran			
Location	33°59′- 35°48′N, and 47°34′-49°36′ E			
Crop period	270 days		210 days	
Planting date	October 2022		November 2022	
Harvesting date	Jun 2023		May 2023	
Cultivar	*Triticum aestivum* L			
Yield (ton/ha)	8.69	6.77	12.2	10.7
Irrigation	Rainwater		Irrigation pump	
